# Evaluation of predictive models to determine total morbidity outcome of feedlot cattle based on cohort-level feed delivery data during the first 15 days on feed

**DOI:** 10.1093/tas/txac121

**Published:** 2022-08-29

**Authors:** L Heinen, P A Lancaster, B J White, E Zwiefel

**Affiliations:** Beef Cattle Institute, Department of Clinical Sciences, College of Veterinary Medicine, Kansas State University, Manhattan, KS 66506, USA; Beef Cattle Institute, Department of Clinical Sciences, College of Veterinary Medicine, Kansas State University, Manhattan, KS 66506, USA; Beef Cattle Institute, Department of Clinical Sciences, College of Veterinary Medicine, Kansas State University, Manhattan, KS 66506, USA; Machine Learning Global Black Belt Team, Microsoft Corporation, Edina, MN 55424, USA

**Keywords:** feeding patterns, machine learning, predictive analytics

## Abstract

Changes in feeding behavior and intake have been used to predict the onset of bovine respiratory disease in individual animals but have not been applied to cohort-level data. Correctly identifying high morbidity cohorts of cattle early in the feeding period could facilitate the administration of interventions to improve health and economic outcomes. The study objective was to determine the ability of feed delivery data from the first 15 days of feed to predict total feeding period morbidity. Data consisted of 518 cohorts (10 feedlots, 56,796 animals) of cattle of varying sex, age, arrival weight, and arrival time of year over a 2-year period. Overall cohort-level morbidity was classified into high (≥15% total morbidity) or low categories with 18.5% of cohorts having high morbidity. Five predictive models (advanced perceptron, decision forest, logistic regression, neural network, and boosted decision tree) were created to predict overall morbidity given cattle characteristics at arrival and feeding characteristics from the first 15 days. The dataset was split into training and testing subsets (75% and 25% of original, respectively), stratified by the outcome of interest. Predictive models were generated in Microsoft Azure using the training set and overall predictive performance was evaluated using the testing set. Performance in the testing set (*n* = 130) was measured based on final accuracy, sensitivity (Sn, the ability to accurately detect high morbidity cohorts), and specificity (Sp, the ability to accurately detect low morbidity cohorts). The decision forest had the highest Sp (97%) with the greatest ability to accurately identify low morbidity lots (103 of 106 identified correctly), but this model had low Sn (33%). The logistic regression and neural network had similar Sn (both 63%) and Sp (69% and 72%, respectively) with the best ability to correctly identify high morbidity cohorts (15 of 24 correctly identified). Predictor variables with the greatest importance in the predictive models included percent change in feed delivery between days and 4-day moving averages. The most frequent variable with a high level of importance among models was the percent change in feed delivered from d 2 to 3 after arrival. In conclusion, feed delivery data during the first 15 days on feed was a significant predictor of total cohort-level morbidity over the entire feeding period with changes in feed delivery providing important information.

## INTRODUCTION

Bovine respiratory disease (BRD) is one of the costliest diseases in the feedlot industry (Salman et al., 1991). Early identification and prompt treatment of the disease can reduce the costs associated with BRD (Booker et al., 2004). Additionally, gastrointestinal diseases, lameness, and other adverse health events negatively impact animal performance, but the prevalence of these other diseases is relatively low. Machine learning has been applied to various fields in the agricultural industry to decrease costs and increase outputs. Predictive modeling techniques have not been heavily studied for use in the beef industry (White et al., 2018). Feedlots collect large volumes of data daily, including feed calls, antimicrobials, and other treatments administered, and various arrival characteristics. These data and more are potential inputs for predictive models that could be used to predict the health outcome of cohorts of cattle. Therefore, the study objective was to evaluate the diagnostic ability of predictive models to determine whether a cohort would have a health outcome of ≥15% total morbidity during the feeding period based on data collected at arrival and feed delivery data for the first 15 days on feed. The hypothesis was that predictive models can accurately predict total feeding period morbidity, and that feed delivery patterns during the first 15 days on feed are important predictors. The objective was to determine the accuracy, sensitivity, and specificity of five predictive models using arrival characteristics and feed delivery data during the first 15 days on feed to correctly identify high (≥15%) morbidity cohorts.

## MATERIALS AND METHODS

Animal Care and Use Committee approval was unnecessary as data were obtained from an existing database of feedlot operational data.

### Data Collection

Daily records for 12,657 cohorts of cattle (1,005,320 animals) were obtained from 10 U.S. feedlots spanning 2018 to 2020. Health event records on an individual animal basis were tied to cohort-level data. A cohort was defined as a group of cattle purchased and managed together but not necessarily housed in the same pen for the entirety of the feeding phase.

### Data Transformation

Data were transformed into the appropriate format and new variables generated before use in predictive models. First, inclusion criteria were applied to the data. Cohorts needed to have complete arrival and feeding data. Feed delivery data beyond 15 days on feed were not included in the dataset, and remaining feed delivery data were adjusted for arrival body weight (percentage of arrival body weight) on a dry matter basis. Any cohorts with missing data were removed. This resulted in removal of many rows of data as several cohorts were rearranged early in the feeding period. Cohorts with extreme values for some arrival characteristics or dry matter intake were also removed to reduce data entry errors. Cohorts were removed if the cohort was designated as a hospital pen, the average arrival weight was less than 182 kg or greater than 545 kg, days on feed was less than 0 or greater than 300, the cohort had not yet been closed out, or dry matter intake as a percentage of average arrival body weight was greater than 5% or less than 0.1% on any given day within the first 15 days on feed. After applying these exclusion criteria, 12,139 cohorts were removed from the dataset. Secondly, data wrangling techniques applied in R software (R Core Team, 2021) were used to create a dataset that consisted of one row per cohort.

**Figure 1.**
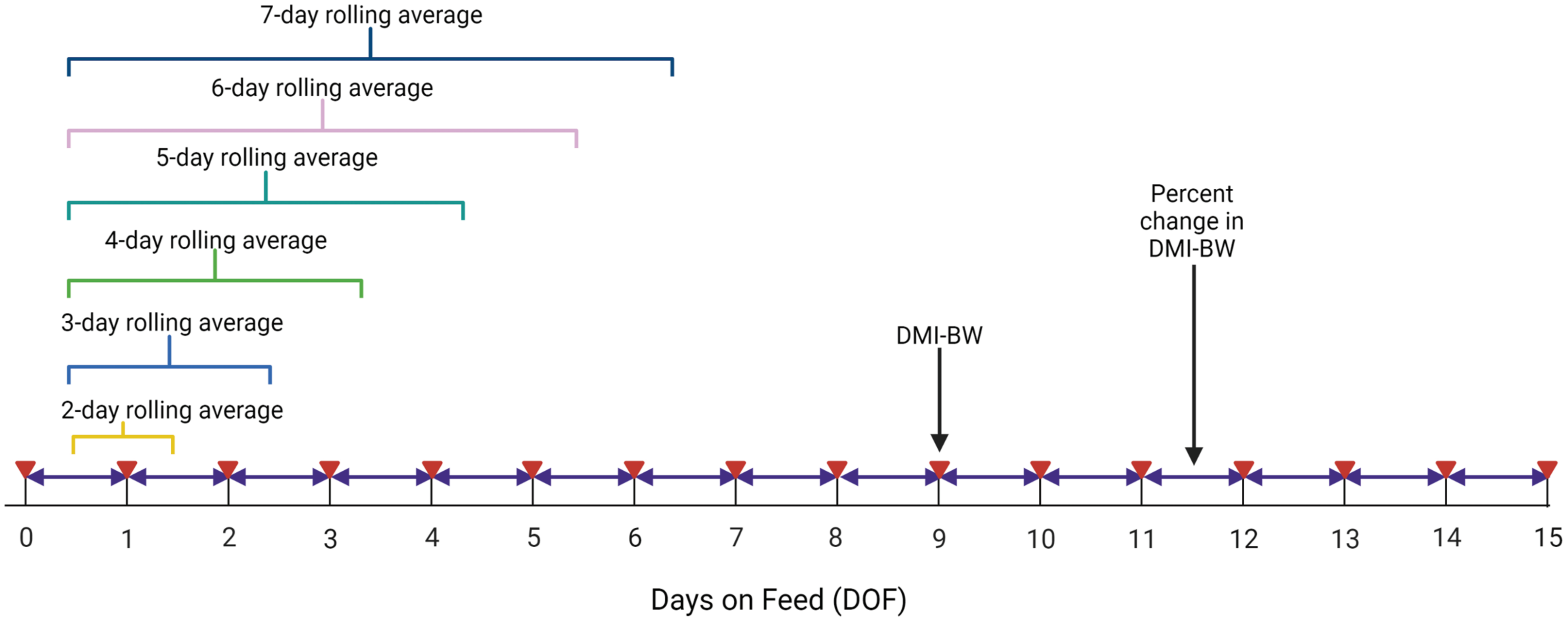
Timeline schematic demonstrating the feed delivery data corresponding to various feeding predictor variables. Triangles indicate data on dry matter delivered as percentage of arrival body weight (DMI-BW). Arrows indicate data on percent change in DMI-BW from day to day.

Several additional variables were created using the existing data to describe feed delivery characteristics such as day-to-day changes in feed delivery and rolling averages of feed delivery using 2-to-7-day time spans. Figure 1 outlines the feeding variables and which days on feed were accounted for in each feeding characteristic variable. Although each variable indicated in the figure is noted only once, feeding variables were calculated for their respective increments throughout the 15-day feeding period of interest. The outcome of interest was captured in a variable called total morbidity category. This variable described the total morbidity (all diseases) of a cohort of cattle during the entire feeding period. It was expressed as a categorical variable in which high morbidity indicated a total morbidity percentage of greater than or equal to 15%. Low morbidity was a total morbidity percentage of less than 15%. Table 1 offers a complete overview of all the variables that populated the dataset. Following data transformation and variable creation, the final dataset consisted of 518 cohorts (56,796 animals) with 18.5% of cohorts having high morbidity.

**Table 1.** Complete overview of variables included in the final dataset to train and test the five predictive models

Variable category	Variable name	Description of variable
Feeding variables	DMI-BW^1^ for day 0 through 15	Feed intake measured by DMI-BW for each day starting on the day of arrival (0) to day 15 (16 total measurements)
	Percent change in DMI-BW from one day to the next for days 0 through 15	Percent change in feed intake measured by DMI-BW between sequential days (15 total measurements)
	2-day increment rolling averages of percent change in DMI-BW	Rolling averages in 2-day increments of percent change in DMI-BW (14 total measurements)
	3-day increment rolling averages of percent change in DMI-BW	Rolling averages in 3-day increments of percent change in DMI-BW (13 total measurements)
	4-day increment rolling averages of percent change in DMI-BW	Rolling averages in 4-day increments of percent change in DMI-BW (12 total measurements)
	5-day increment rolling averages of percent change in DMI-BW	Rolling averages in 5-day increments of percent change in DMI-BW (11 total measurements)
	6-day increment rolling averages of percent change in DMI-BW	Rolling averages in 6-day increments of percent change in DMI-BW (10 total measurements)
	7-day increment rolling averages of percent change in DMI-BW	Rolling averages in 7-day increments of percent change in DMI-BW (9 total measurements)
Arrival characteristics	Arrival date	Date of arrival for the cohort, format: MM/DD/YYYY
	Average arrival weight	Average weight at arrival of the cohort in pounds
	Sex	Sex of the cohort, could be heifer, steer, mixed
	Arrival animal count	Number of animals in the cohort upon arrival
Outcome variable	Total morbidity category	High (≥ 15%) or low (< 15%) based on morbidity for any diagnosis as a percentage of arrival animal count

DMI-BW indicates the feed intake on a dry matter basis given as a percentage of the average arrival weight of the cohort.

Before the model building step occurred, data were split into separate training and testing sets, stratified by the prevalence of outcome of interest. The training set represented 75% of the original dataset while the testing set represented the other 25%. Using the Pipeline Designer function in Azure Machine Learning Studio (Microsoft, 2022), the datasets were used to create five predictive models. The models trained and tested in this study were advanced perceptron, neural network, boosted decision tree, decision forest, and logistic regression. A previous description has been given by Rojas et al. (2022) from our laboratory. Briefly, neural networks and advanced perceptron models can be useful for identifying patterns in operational data, but are difficult to utilize for describing model structure or importance of predictive variables (Rosenblatt, 1957; Zhang, 2010). Boosted decision trees and decision forests are classification models creating a series of splits in data based on attributes to minimize entropy in resulting data subsets (Breiman, 2001; Roe et al., 2005). Logistic regression models are often used for statistical analysis and these algorithms can be created, then used to estimate a probability of even occurrence in a predictive manner (Dreiseitl and Ohno-Machado, 2002). The models selected for investigation in this study are based on previous work conducted by Rojas et al. (2022) and Amrine et al. (2014), as well as the resources available on Azure.

### Model Evaluation

**Figure 2.**
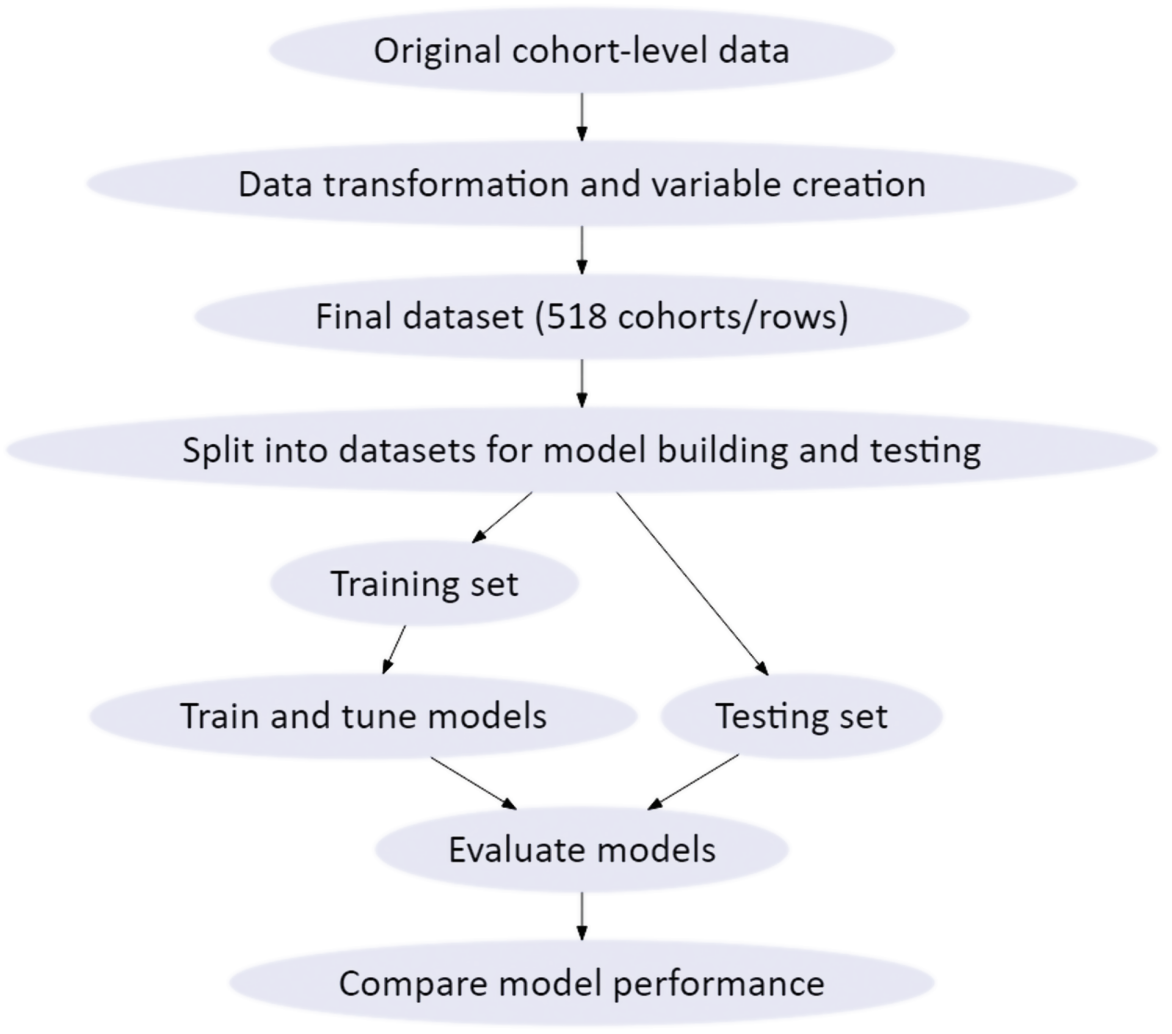
Illustration of data management, and model training and evaluation process.

Finally, test data were used to evaluate model performance. Adjustment of the threshold probability was done manually for each model to maximize F1 score to balance sensitivity and positive predictive value. The metrics used to evaluate the models were accuracy, sensitivity (Sn), specificity (Sp), positive and negative predictive values (PPV and NPV, respectively, and area under the receiver operating characteristics [ROC] curve [AUC]). These values were calculated using the confusion matrices produced by each model run in Azure. Using these metrics, we were able to compare models based on their ability to accurately predict the total feeding period morbidity of a cohort. Figure 2 demonstrates the training and testing process of the predictive models.

## RESULTS AND DISCUSSION

**Figure 3.**
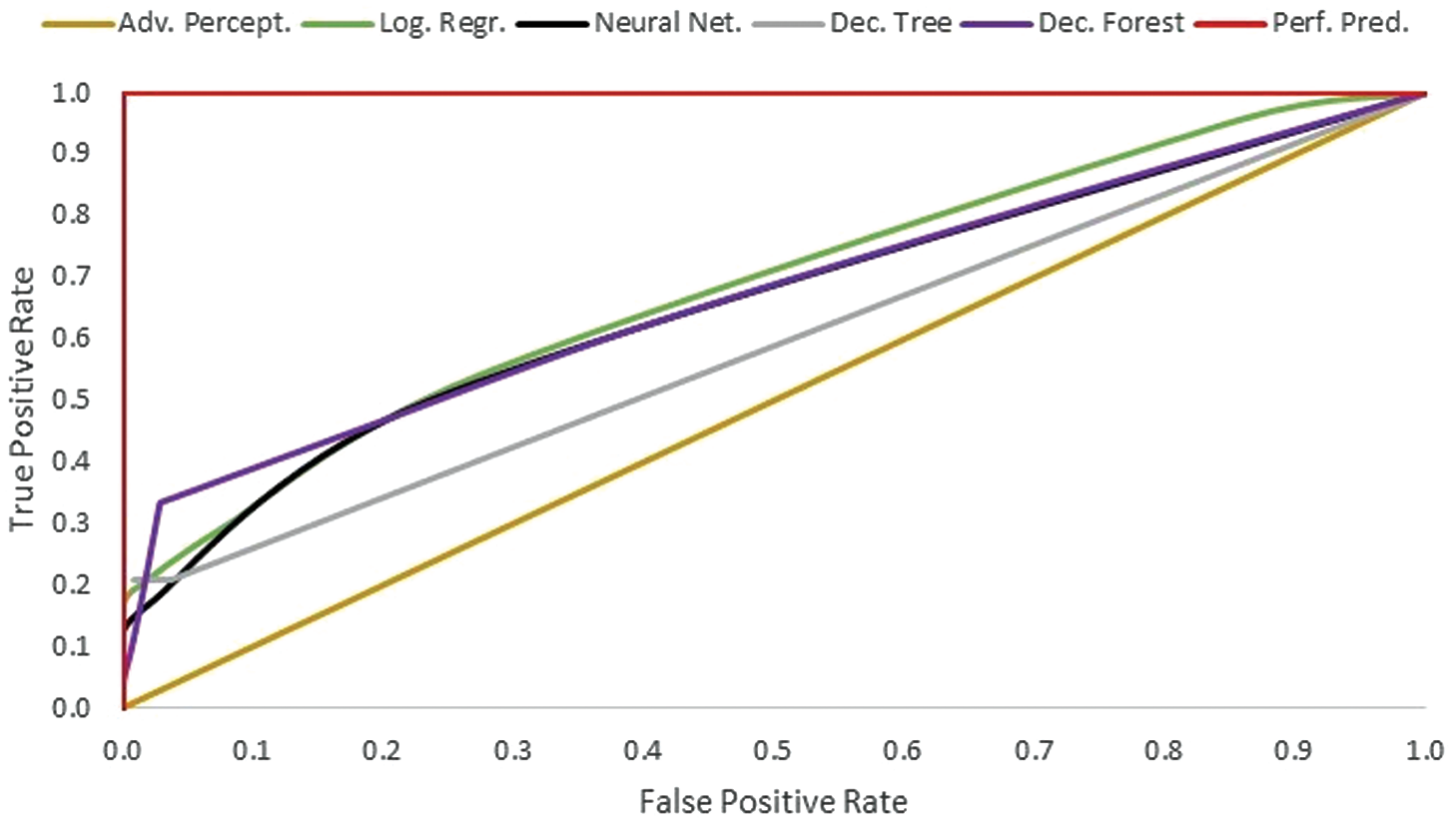
Receiver operating characteristic (ROC) curves for five predictive models trained to predict high (≥15%) morbidity cohorts of feedlot cattle. The five predictive models are Advanced Perceptron, Logistic Regression, Neural Network, Decision Tree, and Decision Forest. Perf. Pred. represents the perfect predictive model.

The ROC curves for the five predictive models are presented in Figure 3. A line closer to a true positive rate of 1.0 and a false positive rate of 0.0 (i.e., top left corner) has greater sensitivity (correctly identify true positives—high morbidity cohorts) and specificity (correctly identifying true negatives—low-morbidity cohorts). Logistic regression, neural network, and decision forest models have similar ROC curves, whereas the decision tree model appears to be slightly less predictive, and the advanced perceptron model has very little predictive ability.

Additionally, the overall accuracy, in addition to the AUC value, support the conclusion that the advanced perceptron model has very poor performance (Table 2). The advanced perceptron model identified all cohorts as high morbidity based on the sensitivity of 100.0%. The logistic regression and neural network models had similar overall accuracy, was well as similar sensitivity, specificity, positive and negative predictive values. The decision tree and decision forest models had the greatest overall accuracy and specificity indicating good ability to identify low morbidity cohorts but had low sensitivity.

**Table 2.** Model evaluation of five predictive models trained to predict high (≥15%) morbidity cohorts of feedlot cattle

Model	AUC^1^	Acc (%)	Sn (%)	Sp (%)	PPV	NPV
Advanced perceptron	0.653	18.5	100.0	0.0	0.18	—
Logistic regression	0.675	67.7	62.5	68.9	0.31	0.89
Neural network	0.691	70.0	62.5	71.7	0.33	0.89
Decision tree	0.691	78.5	29.2	89.6	0.39	0.85
Decision forest	0.671	85.4	33.3	97.2	0.73	0.87

AUC, area under the receiver operator characteristic (ROC) curve; Acc, overall accuracy; Sn, sensitivity, ability to predict high morbidity cohorts; Sp, specificity, ability to predict low morbidity cohorts; PPV, positive predictive value, probability that predicted high morbidity cohorts are truly high morbidity cohorts; NPV, negative predictive value, probability that predicted low morbidity cohorts are truly low morbidity cohorts.

Previous work from our lab (Amrine et al., 2019) reported that predictive models with greater specificity had lesser sensitivity when using sale barn and arrival characteristics to predict BRD in the first 14 days on feed. The “best” model depends upon the goals of the feedlot manager. If the goal is to accurately identify low morbidity cohorts, then a model with the greatest specificity would be deemed the ‘best’ model. Conversely, if the goal is to accurately identify high morbidity cohorts, then a model with the greatest sensitivity would be deemed the ‘best’ model. Accurate identification of high morbidity cohorts could lead to actions that allow changes in risk management, frequency of disease monitoring, and other interventions. However, if the model misclassifies low morbidity cohorts as high morbidity cohorts, then valuable resources would be wasted. The decision forest model had a low sensitivity, only accurately classifying 33% of high morbidity cohorts, but based on the high PPV, this model had a high probability of being correct when it did classify a cohort as high morbidity. Thus, the decision forest model would not identify all of the high morbidity cohorts, but if potential interventions were implemented based on this model little resources would be wasted.

Several previous studies (Sowell et al., 1999; Buhman et al., 2000; Quimby et al., 2001; Moya et al., 2015; Wolfger et al., 2015; Jackson et al., 2016) have indicated that feed intake, feeding behavior, and drinking behavior are predictive of onset of BRD in individual cattle. The onset of BRD can be predicted 4 to 7 days before clinical signs can be observed using feeding and drinking data (Buhman et al., 2000; Moya et al., 2015; Wolfger et al., 2015; Jackson et al., 2016). Feeding behavior has predicted BRD with an overall accuracy of 84% to 89% and positive predictive value of 85% to 96% (Quimby et al., 2001). Wolfger et al. (2015) indicated that feed behavior correctly predicted 81% of BRD cases and 77% of healthy animals 3 days prior to clinical signs; adding feed intake did not improve these predictions. Similarly, Moya et al. (2015), using pattern recognition techniques, reported that feeding behavior predicted BRD with good sensitivity (58% to 83%) and specificity (67% to 100%) for some of the models with the best overall accuracy.

From the Microsoft Azure platform, feature importance could be obtained for the decision tree, decision forest, and logistic regression models. In each of these models, feeding data were among the top five predictors, and the percent change in feed delivery from days on feed (DOF) 2 to 3 was one of the most important predictors. Other predictors were percent change in feed delivery from DOF 4 to 5 and from DOF 8 to 9, and rolling average in percent change in feed delivery from DOF 7 to 10. These results indicate that alterations in feeding patterns very early in the feeding period are predictive of total morbidity, which may allow interventions to mitigate disease progression in the cohort.

In commercial feedlots, cattle are managed as pens except for individual animal treatment of disease. Tracking individual animal feeding behavior is not practical nor cost-effective in a commercial feedlot. However, commercial feedlots collect real-time data that can be used to make pen-level management decisions. The results of this study and the previous discussion indicate that feeding data can be used to predict morbidity in cattle. In our study, we looked at total morbidity, but BRD accounted for 65% of the total treatments. The predictive models in this study were somewhat predictive of total cohort level morbidity indicating that feeding data can be used to predict morbidity in pens of cattle, which could lead to improved management of disease in feedlots. However, in the current study, feed delivery data were used as predictors in the models, which do not account for feed refusals. Based on previous data (Jackson, 2016), feed intake decreases prior to clinical signs of BRD and thus in our case, a decrease in the feed delivered is likely indicative of significant feed refusals and decreased feed intake for the day prior. Although, an increase in feed refusals could also be due to several other factors such as weather, removal of cattle from the pen, etc., which cannot be ascertained from our current dataset.

## CONCLUSION

Feed intake and feeding behavior data are predictive of BRD in individual animals, and feed delivery data are predictive of total morbidity in commercial feedlot pens. Predictive analytics is a valuable tool that can be used to convert feedlot operational data into animal health management decisions. Future research should evaluate the ability of feed delivery to predict specific diseases (BRD, bloat, etc.), and combine feed delivery data with other data types to improve the prediction of morbidity in feedlot cattle.
